# Cytoreduction surgery (CRS) with hyperthermic intraperitoneal chemotherapy (HIPEC) as treatment choice of metastatic Urachal carcinoma

**DOI:** 10.1016/j.ijscr.2024.109467

**Published:** 2024-03-04

**Authors:** Giorgio Micheletti, Vincenzo Ricchiuti, Ludovico Carbone, Noemi La Francesca, Roberto Petrioli, Daniele Marrelli

**Affiliations:** a Unit of Surgical Oncology, Department of Medicine Surgery and Neurosciences, University of Siena, Italy; b Department of Medical Biotechnology, University of Siena, Italy; c Unit of Medical Oncology, Department of Medicine Surgery and Neurosciences, University of Siena, Italy; d Kidney Transplant Unit, Department of Medicine Surgery and Neurosciences, University of Siena, Italy

**Keywords:** Urachal carcinoma, Cytoreduction surgery, Hyperthermic intraperitoneal chemotherapy, Peritoneal metastasis, Peritoneal Cancer Index, Case report

## Abstract

**Introduction:**

Urachal carcinoma accounts for approximately 0.01 % of all adult malignancies and 1 % of bladder cancers. Its prognosis remains poor, with a 5-year overall survival rate of less than 50 %.

**Presentation of case:**

A 51-years-old black female, affected by peritoneal malignancies from urachal carcinoma, underwent multiple surgical cytoreduction (CRS) and hyperthermic intraperitoneal chemotherapy (HIPEC) with different chemotherapy regimen, alternating with intravenous chemotherapy. Thirty-two months recurrence-free survival was registered, and overall survival was more than 5 years.

**Discussion:**

Our case suggests the importance of rigorous follow-up with both tumor marker testing (CEA) and imaging studies. Optimal debulking surgery plays a pivotal role in controlling primary and recurrent disease. The use of combined intraperitoneal and intravenous chemotherapy may have contributed to her long-term survival.

**Conclusion:**

CRS and HIPEC combined with intravenous chemotherapy may be potential candidates for treating patients with urachal carcinoma with peritoneal metastases. Our patient is a challenging case in daily surgical practice.

## Highlights

• Carcinoma of the urachus has an aggressive behavior. The mucinous type may invade the peritoneum within a few months.
• Metastatic stage may benefit from surgical and bidirectional therapy, combining intraperitoneal and systemic chemotherapy.
• Although recurrence are common, cytoreduction surgery and intraperitoneal chemotherapy improve disease-free survival.

## 1 Introduction

Urachal carcinoma (UC) is a rare malignant tumor [1,2], first described by *Hue* and *Jacquin* in 1863. The urachal [3,4] duct is a conduit connecting the bladder to the allantoid, the lumen of which is obliterated in the median umbilical ligament stretched from the umbilicus to the bladder dome during fetal development. Autopsy studies have shown the persistence of partial and non-partial patency in approximately 1/3 of adults sometimes leading to the occurrence of malignant evolution [1,2]. Patients with UC have a poor prognosis [5], with a 5-year overall survival not exceeding 50 %. After cancer invade the bladder, cancer cells tend to break away from the primary site to lung, liver and bone [1,2].

Adenocarcinoma represents more than 80 % of all UC, and the mucinous variant is the most common histologic subtype, containing an abundance of mucin with clusters of floating tumor cells. It has an aggressive behavior [6], often spreads to the peritoneal cavity and shows resistance to conventional treatments [7]. Other histotypes are relatively rare and include tumor not otherwise specified (NOS), signet ring cell (SRC), squamous cell, urothelial carcinoma and sarcoma.

Extended partial cystectomy or radical cystectomy represent the core of treatment. Negative surgical margins and uninvolved lymph nodes are main prognostic factors. Otherwise, evidence on the effectiveness of perioperative therapies in the management of metastatic disease is still controversal.

This paper reports a case of diffuse peritoneal metastasis of mucinous UC treated with cytoreductive surgery (CRS) plus hyperthermic intraperitoneal chemotherapy (HIPEC) followed by bidirectional therapy, combining intraperitoneal and systemic chemotherapy.

The present work has been reported according to the *SCARE* criteria [8].

## 2 Presentation of case

The 51-year-old black woman's medical history started in August 2016; she presented with spontaneous leakage of gelatinous material from the umbilicus. Her other medical history included hysterectomy, performed due to postpartum hemorrhage with fibromatous uterus, arterial hypertension, and hypercholesterolemia.

**Fig. 1:**
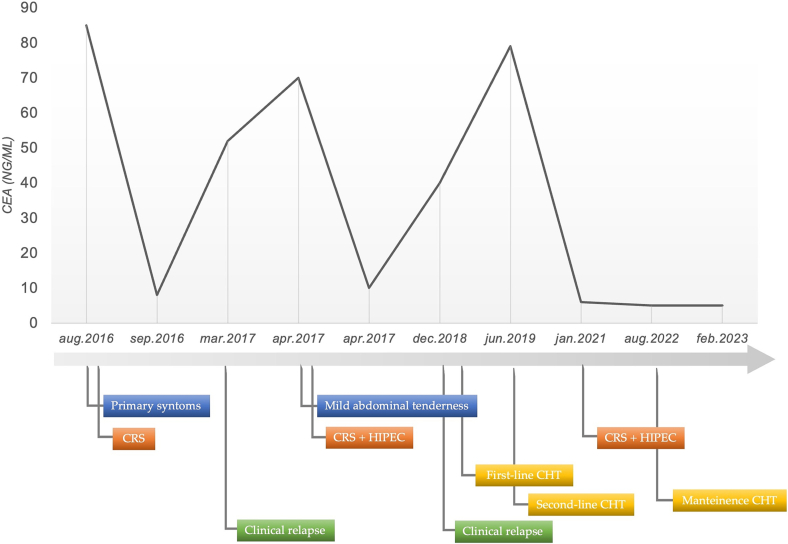
Trends in the serum Carcino-Embryonic Antigen (CEA) values.

The baseline CEA level was 85 ng/ml (normal range 0 to 2.5 ng/ml) (Fig. 1). Magnetic resonance imaging (MRI) showed a fistulized nodular formation through the skin. This lesion revealed multiple septa, cm 5 × 3 in diameter with liquid-corpuscular contents, connected with the skin through the rectus abdominis. No other lesions in the main abdominal parenchyma were reported. She underwent the surgical removal of a paraumbilical cystic neoformation (en-bloc with the urachus) and its insertion on the bladder, umbilicus, a portion of adherent omentum and muscle fascia.

Histopathologic examination emphasized the presence of a mucinous adenocarcinoma cystic tumor covered by intestinal epithelium with low-grade atypia arising from the urachus. It showed low malignant potential and negative microscopic margin (*Sheldon* stage II) [3] (Table 1). Serum carcino-embryonic antigen (CEA) tended to normalize.

**Table 1:** Different staging system in Urachal carcinoma.

*Sheldon* staging system	
I	No invasion beyond the urachal mucosa
II	Invasion confined to the urachus
IIIA	Local extension into the bladder
IIIB	Local extension into the abdominal wall
IIIC	Local extension into the peritoneum
IIID	Local extension into viscera other than the bladder

*Mayo* staging system	
I	Confined the urachus and/or bladder
II	Extension beyond the muscular layer of the urachus and/or the bladder
III	Infiltration to the regional lymph nodes
IV	Infiltration to non-regional lymph nodes or other distant sites

*TNM* staging system	
T1	Tumor invades the subepithelial connective tissue
T2a	Invasion of the deep muscle (outer half) layer of the urachus of bladder
T2b	Invasion of the superficial muscle (inner half) layer of the urachus of bladder
T3	Invasion of the perivisceral soft tissue, prostate, uterus, or vagina

An interdisciplinary team planned her radiologic follow-up. Two months later, MRI showed minimal perihepatic effusion. Exploratory laparoscopy performed 10 weeks after surgery described millimeter-sized pelvic peritoneal mucus collection. In March 2017 the patient underwent delayed MRI which showed disease recurrence in the right subdiaphragmatic space and left hypochondrium. A thoracic-abdominal computed tomography (CT) scan revealed a nodular formation at the apical segment in the left lower lobe of the lung, which was not clearly interpreted and warranted follow-up. The serum CEA level was 51.6 ng/ml.

**Fig. 2:**
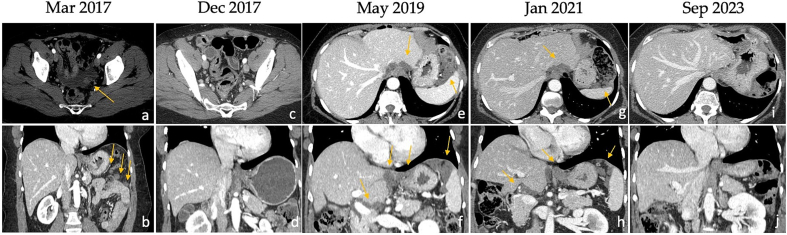
CT scan: In March 2017 the first relapse of the disease was assessed at our radiology department. At CT, on the axial plane (a), a small amount of free fluid and peritoneal nodules are visible, quite evident also along the left parietocolic gutter in the coronal plane (b). After CRS and HIPEC (April 2017) the CT in December 2017 (c, d) was completely negative. In May 2019 the patient had a second relapse and peritoneal irregular thickening consisting of confluent nodules is detectable near the spleen, under the left diaphragm and along the hepatic capsule (e, f). In January 2021 only a small reduction was visible (g, h) but it was considered enough to attempt a new treatment by CRS and HIPEC. At the most recent follow-up CT scan, in September 2023, there are no signs of relapse.

**Fig. 3:**
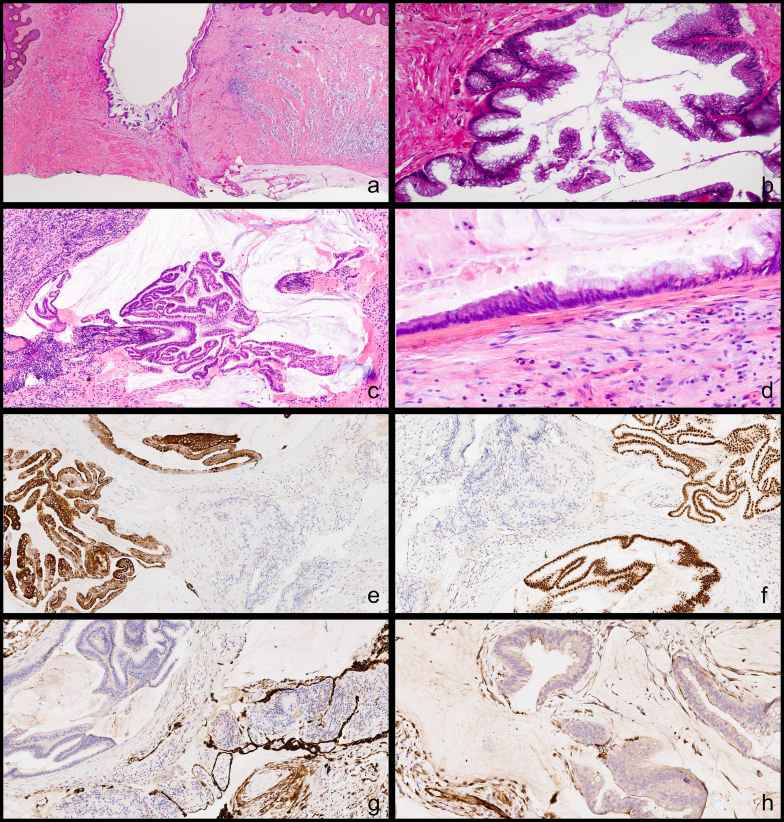
Morphology and immunophenotype of a mucinous cystic tumor of low malignant potential: a: Evidence of transition from benign urothelium (upper portion) to atypical mucinous epithelium (lower portion) in a paraumbilical skin fistulizing multicystic tumor; b: At higher magnification, cysts were lined by cylindrical mucinous epithelium tufted and proliferating with low-grade atypia; c: Peritoneal nodules were composed by mucinous lakes focally lined by mucinous proliferating epithelium, floating in the mucinous material; d: On the right of the image, apical intracytoplasmic mucin deposits in the lining epithelium are noted; e, f: The neoplastic cells stained positive with cytokeratin 20 (e) and showed nuclear expression of CDX2 (f); g, h: The cytoplasmatic cytokeratin 7 (g) and the nuclear WT1 (h) were normally expressed by the mesothelial cells while the neoplastic cells resulted were negative.

Thus, the patient was referred to our hospital in March 2017. She had mild abdominal tenderness only on deep palpation. An additional increase in CEA levels was recorded and thoracic-abdominal CT highlighted a dimensional increase above 20 % of peritoneal carcinosis multiple implants with pathological lymph nodes at right cardiophrenic angle (Fig. 2). A definitive CRS was performed; Peritoneal Cancer Index (PCI) and Completeness of Cancer Resection (CCR) score were calculated. Disease localizations were identified at the hepatic Glisson's capsule, hepatoduodenal and hepatogastric ligaments, diaphragm, greater curvature of the stomach, greater omentum, pelvic peritoneum, jejunal and distal ileum, their mesenteries and anterior rectal wall. The removal of all macroscopic disease sites and the parietal peritoneum, omentectomy, cholecystectomy, appendectomy, oophorectomy, and the removal of uterine remnants at the vagina were performed, without bowel resections. Calculated PCI was 13 and CCR score 1, as optimal debulking was performed [6]. Peritoneal cytology resulted positive. The HIPEC was performed with the closed technique and perfusion of cisplatin and mitomycin C (100 mg/m^2^ and 20 mg/m^2^ respectively, with 1.94 m^2^ of total body surface area) for 1 hour at 41–43 °C with a mean flow of 700 ml/min. The postoperative course was uneventful. The final histopathologic examination described recurrence of G2 mucinous urachal adenocarcinoma, intestinal subtype with medium degree of differentiation (CDX2+, CK20+, CK7-, WT1-). All 8 lymph nodes harvested were not involved (N0) (Fig. 3). The post-operative CEA value dropped to 9,4 ng/ml. After a multidisciplinary team evaluation, no adjuvant therapy was administered, while close follow-up was planned with both imaging examinations (MRI or CT when necessary) and tumor marker testing (CEA).

The patient remained progression-free for 20 months. New peritoneal recurrence in the surface of the left diaphragm, gastrosplenic ligament, omental bursa, hepatic hilum, and laparotomic wound was noted on follow-up CT in December 2018. CEA value was 38.7 ng/ml (Fig. 2). The patient started a systemic chemotherapy to control the recurrence of the disease according to the XELOX scheme (capecitabine plus oxaliplatin, 6 cycles).

In June 2019, due to poor follow-up response and elevated CEA, the patient was started on second-line FOLFIRI (leucovorin calcium, 5-FU, and irinotecan) and Bevacizumab was taken. Imaging studies documented progressive reduction of peritoneal metastases.

In January 2021 the peritoneal burden was further reduced, and CEA dropped to 5.9 ng/ml. A total of 42 cycles of systemic second-line chemotherapy were administered. The patient underwent a second CRS and HIPEC with cisplatin and mitomycin C (100 mg/m^2^ and 20 mg/m^2^ respectively, with 2.00 m^2^ of total body surface area). PCI was 11 and CCR score 0. There were not postoperative complications. Adjuvant chemotherapy with Bevacizumab was started for twelve cycles once a month until June 2022.

At the last follow-up in September 2023, CEA levels (7.6 ng/ml) and a new CT scan confirmed a recurrence-free status (Fig. 2). Therefore, overall survival at last follow-up was 86 months.

## 3 Discussion

Given the rarity of UC, evidence-based recommendations for the appropriate management of localized and metastatic forms are lacking.

A) Localized or locally advanced cancers benefit from upfront removal of the urachus, umbilicus and extended partial or total cystectomy. Partial cystectomy has been shown to have non-inferior oncologic safety compared to radical cystectomy [1]. While the role of pelvic lymphadenectomy is still debated, *Duan* defined pelvic lymphadenectomy as “lymph node sampling from the obturator, internal iliac, and external iliac lymph node stations” [9]. Since positive lymph nodes have been shown to worsen prognosis [1,2,9,10], several authors recommend pelvic lymphadenectomy to improve the staging of the disease [1,2,9,10]. Sheldon stage ≥ IIIB, distant metastases and positive surgical margins [10,11] are negative prognostic factors. Although mucinous type, our case showed no additional factors of recurrence. Radiotherapy is ineffective [1,2,12].
B) In unresectable and/or metastatic forms, chemotherapy with cisplatin has been shown to improve progression-free survival compared with non‑platinum based regimens [2,15].

Biological studies have recently identified molecular features, such as K-RAS, B-RAF and N-RAS mutations [13], that are shared with the colorectal adenocarcinoma. Based on these findings, 5-FU and platinum-derived drugs have shown optimal survival outcomes [1,2,12].

In 1985, *Logothetis* first demonstrated the efficacy of the combination of 5-FU, doxorubicin, mitomycin and cisplatin-based therapy, reporting a case of 11 months disease-free survival [14]. The combination of 5-FU and cisplatin has been associated with improved survival, compared to cisplatin or 5-FU alone [2]. Addition of 5-FU, leucovorin, gemcitabine and cisplatin showed radiographic response rates of 30–40 %. Other cases showed nearly complete disease regression with the modified FOLFOX6 scheme (leucovorin, 5-FU, and oxaliplatin) [15]. *Kume* reported a case of multi-therapiy chemoresistant UC, that was sensitive to irinotecan, resulting in a decrease of CEA levels (from 98.3 to 38.7 ng/ml) and a 60 % reduction in lung metastases [16]. Encouraging results with the FOLFIRI regimen have also been described. The combination of FOLFOX or FOLFIRI and Bevacizumab is commonly used in the treatment of advanced colon cancer to control peritoneal relapse progression. *Kanamaru* reported a case of a lung and mediastinal metastases from UC; the patient initially underwent upfront surgery, whereas peritoneal recurrence occurred four years later. The first line of cisplatin-based drugs and S-1 did not result in disease regression, while a second line of FOLFIRI plus Bevacizumab resulted in 12 months of progression free survival [17]. A mutation in the EGFR gene suggests a possible use of other monoclonal antibodies (Cetuximab or Panitumumab) [1,18].

The HIPEC is a valid treatment option, as the UC has histopathologic and molecular features overlapping with gastrointestinal carcinoma. In *Krane*'s series, a total of six patients underwent CRS and HIPEC with mitomycin, resulting in 27 and 13 months overall and disease-free survival respectively [19]. Similarly, *Yang Liu* described 27.5 months disease-free survival after CRS and HIPEC with mitomycin C and cisplatin [20]. After peritoneal recurrence, a second HIPEC was performed to improve progression-free survival and minimize peritoneal cancer complications.

## 4 Conclusion

UC is a rare disease with poor prognosis. Advanced forms may benefit from complete surgical resection and chemotherapy. “*Satis quod sufficit*”. Due of the high rate of recurrence, regular follow-up and CEA measurement are highly recommended.

## Ethical approval

Ethical approval was not sought from our institution, as this is a case report.

## Funding

This research did not receive any specific grant from funding agencies in the public, commercial, or not-for-profit sectors.

## Author contribution

Conceptualization: D.M.; Data curation: V.R.; Investigation: G.M.; Methodology: L.C. and N.L.F.; Project administration: D.M.; Supervision: R.P.; Validation: D.M.; Visualization: N.L.F.; Roles/Writing - original draft: G.M. and L.C.; and Writing - review & editing: D.M. and R.P.

## Guarantor

Prof. Dr. Daniele Marrelli.

## Research registration number

N/A.

## Conflict of interest statement

The authors have no conflicts of interest.
